# GPCR_LigandClassify.py; a rigorous machine learning classifier for GPCR targeting compounds

**DOI:** 10.1038/s41598-021-88939-5

**Published:** 2021-05-04

**Authors:** Marawan Ahmed, Horia Jalily Hasani, Subha Kalyaanamoorthy, Khaled Barakat

**Affiliations:** 1 Faculty of Pharmacy and Pharmaceutical Sciences, University of Alberta, 116 Street & 85 Avenue, Edmonton, AB T6G 2R3 Canada; 2 Department of Chemistry, University of Waterloo, Waterloo, N2L 3G1 Canada; 3 Li Ka Shing Institute of Virology, University of Alberta, 116 Street & 85 Avenue, Edmonton, AB T6G 2R3 Canada

**Keywords:** Computational biology and bioinformatics, Drug discovery, Chemistry

## Abstract

The current study describes the construction of various ligand-based machine learning models to be used for drug-repurposing against the family of G-Protein Coupled Receptors (GPCRs). In building these models, we collected > 500,000 data points, encompassing experimentally measured molecular association data of > 160,000 unique ligands against > 250 GPCRs. These data points were retrieved from the GPCR-Ligand Association (GLASS) database. We have used diverse molecular featurization methods to describe the input molecules. Multiple supervised ML algorithms were developed, tested and compared for their accuracy, F scores, as well as for their Matthews’ correlation coefficient scores (MCC). Our data suggest that combined with molecular fingerprinting, ensemble decision trees and gradient boosted trees ML algorithms are on the accuracy border of the rather sophisticated deep neural nets (DNNs)-based algorithms. On a test dataset, these models displayed an excellent performance, reaching a ~ 90% classification accuracy. Additionally, we showcase a few examples where our models were able to identify interesting connections between known drugs from the Drug-Bank database and members of the GPCR family of receptors. Our findings are in excellent agreement with previously reported experimental observations in the literature. We hope the models presented in this paper synergize with the currently ongoing interest of applying machine learning modeling in the field of drug repurposing and computational drug discovery in general.

## Introduction

With an annual research spending that often exceeds $100 billion USD per year^1^, the pharma industry is still striving for novel ideas to discover new drugs to treat challenging diseases. Not surprisingly, the high attrition rate, even at the final stages of the drug development process, is a substantial risk factor for this industry pushing it toward adopting very different business models geared toward cost-saving. Currently, numerous pharmaceutical companies are using the opportunity offered by repurposing existing drugs as a novel strategy to improve their research & development (R&D) cost efficiency^1,2^. This is because drug repurposing can significantly shorten the amount of time and cost needed to identify and develop a new drug^3,4^. In a research survey published in 2015, Kesselheim et al. showed that approximately 35% of the FDA approved drugs in the interval between 1984 and 2009 were repurposed from products that were invented for other medical indications^2^.

The concept behind drug repurposing is simple and relies on the fact that drug molecules often interact with multiple biological targets. In this framework, the observed therapeutic/side effects of a typical drug molecule result from the collective pharmacological footprints generated from these interactions^5–7^. For example, the well-established pharmacological activities of resveratrol, a trans-stilbene natural product, which exists in grapes, in many diseases have been attributed to its ability to interact with many biological targets^8^. Resveratrol exerts cardioprotective^9^, neuroprotective^10^, anti-viral^11^, as well as other pharmacological activities^8^. A second example is the discovery of the antiviral activity of raloxifene against the Ebola virus. Raloxifene has been originally identified for its ability to reduce the risks of developing breast cancer and osteoporosis^12,13^. A third example is sildenafil (Viagra), which was originally developed to treat cardiovascular problems. The drug was later discovered to enhance the erectile dysfunction in male patients and is currently one of the best-selling drugs in the world^14^. The list of successful examples of drug repurposing goes on and on, and this outstanding success emphasized drug repurposing as an effective research arm against emerging life-threatening diseases^15^. This has been clearly shown in recent studies against COVID-19 (e.g. hydroxychloroquine and remdesivir). A comprehensive repository of repurposed drugs can be found in the RepurposeDB database, which contains drug-repurposing data for 253 medications (as of July 2019) and 1125 diseases^16^.

Despite the significant progress in experimental approaches for drug repurposing screening, these approaches are challenged by their resource-intensive nature. Therefore, there will always be a need to develop better and faster computational models and databases that can identify potential drug candidates suitable for repurposing^17^. This is supported by the recent advances in high performance computing, proteomics, pharmacogenomics, and data mining techniques, which enabled novel strategies to repurpose approved and investigational drugs. An example of such strategies is the protocol developed by Eric Wen Su to mine the adverse events data deposited in the ClinicalTrials.gov database^18^. Another example is the work of Chakraborti et al., who used the evolutionary relationship between established targets and the proteins of the pathogenic microorganisms to suggest a few FDA approved drugs that can be repurposed as anti-infective agents^19^. These two examples adopted a purely target-based protocol to identify drug-repurposing instances. However, given the complex landscape of the evolutionary relationships between biological macromolecules, ligand-based computational modeling approaches seem to be more practical.

Ligand-based models are developed using the molecular representations of the input molecules. These representations include the molecular physicochemical properties or molecular fingerprints of a given molecule^20,21^. The ultimate goal of a ligand-based model is to map these molecular representations to the molecular properties of interest using a well-established set of computationally intensive algorithms. In this context, machine learning (ML) stands on top of all ligand-based methods as a promising drug repurposing tool^22,23^. A promising example of using ML models for drug repurposing is the Computational Analysis of Novel Drug Opportunities (CANDO) platform, which aims at proposing every existing drug or compound for every disease^24^. In their most recent version, CANDO developers claimed a success rate of 19% at the top 10 cut-off, compared to 11.7% for the previous version, and 2.2% for a random control^25^. Furthermore, the CANDO developers also pointed out that decision tree-based ML algorithms combined with the ECFP4 molecular fingerprints yielded the best performance overall.

The current study implements a ligand-based ML protocol to study the possibility of repurposing small molecule ligands against G protein coupled receptors (GPCRs). GPCRs represent one of the most important families of drug targets and from a market share perspective, it was estimated that ~ 700 of all approved drugs (35%) are targeting GPCRs^26^. In this context, the aims of the current study are two-fold. First is model development and second is the application of these models in a real-world scenario. Towards the first objective, we developed 15 supervised ML models to classify known GPCR ligands according to their GPCR targets. These models were trained and validated using the molecular association data of drug/drug-like molecules against known GPCRs. It is important to note that the overall design of our work is entirely different from a recently published study by Seo et al.^27^ which also aimed at classifying known GPCR ligands. In their study, Seo and his collogues limited their chemical space to the known Lipinski chemical roles (e.g. molecular weight less than 500). They also included receptor-based descriptors (e.g. amino acid sequence information) along with Lipinski-ligand-based descriptors, such that the target variable will be binary (1 if there’s an interaction, 0 otherwise). As the ultimate goal of our study was to look for hidden repurposing opportunities within known drugs and to use the developed ML models to blindly screen compound databases for potential GPCR ligands, including receptor-based descriptors within the model-building pipeline will limit the applicability of the final models. This has been further discussed by Peon et al.^28^, who pointed out that in the contrary from target-centric models, ligand-centric models have the clear advantage of being applicable to any target where at least a single ligand is known for this particular class label. Therefore, we decided to rely mainly on the ligand-based descriptors. Furthermore, training the ML models represents an additional difference between the current work and Seo’s study. To reduce the computational cost, Seo et al. divided their dataset into 10 smaller training datasets and trained the classifiers on these individual datasets. In our implementation, however, we trained our classifiers using the full (training) dataset and tested the performance of the models on a test set. This process was repeated five times to include the statistical variations on the test scores. Our reported final scores are averaged across the prediction outputs on the test datasets of these five independent runs. Although, classical n-fold cross-validation was impractical given the large size of our dataset, it was performed on a reduced sample of the dataset to tune the hyperparameters of the adopted ML models.

To achieve the second goal of the current study and to test our findings in a real-world scenario, we have applied our models to the Drug-Bank dataset of approved, investigational and obsolete drugs. This case study provides a concrete example for integrating ligand-based ML modeling with conventional structure-based methods, such as docking and MD simulations. Results from these simulations are further discussed in light of recent experimental pieces of evidence. We finally highlight the potential pitfalls that may arise as a result of inherent limitations of the adopted data acquisition and ML modeling approaches.

## Methods

### Dataset selection

In order to develop robust models to tackle the current problem, the first step was to collect a suitable dataset with several examples to train these models. To ensure an adequate coverage of the entire chemical space of drug-like molecules, we decided to use the GPCR-Ligand Association (GLASS) database^29^ from the Zhang lab (University of Michigan, USA). GLASS is a free, curated database of experimentally validated GPCR-ligand interaction data. As of February 2019, this database included experimentally measured binding affinities for 562,871 unique GPCR-ligand interaction records, comprising ~ 342,539 unique ligands and 3056 unique GPCRs. The data from the GLASS database can be downloaded as tab-separated value (TSV) files. To the best of our knowledge, GLASS represents the largest curated repository available for experimentally validated GPCR-ligand association data.

### Adopted protocol for ligand/receptor filtering, data cleaning and pre-preprocessing

The entire process of data cleaning and preparation was performed using the Pandas library in the Spyder Python IDE (Anaconda). The following three TSV spreadsheets were retrieved from GLASS:

1. *Ligands.tsv*: Contains ligands-related information, such as the ligands’ names, canonical SMILES, InChI key, XLogP, and the number of hydrogen bond donors/acceptors.
2. *GPCR_targets.tsv*: Contains information on GPCR targets, including GPCR names, FASTA sequences, Uniprot IDs, Gene names and species.
3. *Interactions_actives.tsv*: Contains assay records of the ligands against the corresponding GPCR targets.

The three files were merged into a single spreadsheet and were prepared for building the ML models after performing a few data cleaning steps. The data cleaning workflow included:

1. Removing spaces and special characters from columns headers.
2. Removing any redundancies in the GPCR targets by grouping the GPCR subtypes into a single type. For example, all Alpha-adrenergic receptor subtypes (e.g. Alpha-1A adrenergic receptor, Alpha-1B adrenergic receptor, Alpha-1D adrenergic receptor, Alpha-2A adrenergic receptor, Alpha-2B adrenergic receptor & Alpha-2C adrenergic receptor) were merged into a single type, i.e. Alpha-adrenergic receptor, and so on.
3. For ligands that are active against multiple GPCR types, only the record with the highest affinity of each ligand was kept, such that the final dataset contains a single activity record for each unique ligand.
4. Receptors possessing less than 25 assay records were excluded.
5. We focused on the subset of ligands that exhibited an acceptable level of drug-likeness. Therefore, ligands with Molecular Weight < 100 Da or > 900 Da were excluded. Also, ligands with very high (> 10) or very low (< − 4) XlogP values were excluded from the final dataset. However, we did not strictly follow Lipinski's rules of five (Log P < 5 and molecular weight < 500)^30,31^ to select our focused set of compounds. This was to comply with the fact that many drug-like molecules violate the Lipinski recommended range of drug-likeness. Especially, drugs that are of natural origins, drugs that exhibit extensive first pass metabolism as well as non-oral drugs.

After completing the data cleaning protocol, the final dataset contained the assay records of 148,128 unique ligands against 88 unique GPCR targets.

### Molecular featurization using the DeepChem library

Molecular featurization involves generating a fixed-length vector of features to describe the input molecules. This fixed-length vector has been populated by (i) molecular descriptors (physicochemical properties; experimental or calculated), and (ii) molecular fingerprints. In the current study, we have explored both featurization methods, (i.e. molecular descriptors and molecular fingerprints). Both featurization methods were accessed through their implementations in the RDKit module of the open-source chemical informatics platform DeepChem^32^. For the molecular descriptors, we have calculated an extensive set of 111 3D molecular descriptors and appended them to the input molecule Pandas dataframe. The list of the exploited molecular descriptors is given in the Supplementary Information S1. For the molecular fingerprints, we have computed depth-2 Extended-connectivity fingerprints (ECFP6) hashed to 2048 bits and a radius of 4; which gives rise to a 2048-length bit vector for each input molecule. To select the best combination of modeling algorithm and featurization method, all developed ML models were separately trained on both molecular representations methods and compared (all details related to model training, validation and assessment are discussed below). As a baseline, we have trained the same ML models against the original four descriptors retrieved from the GLASS database. These descriptors are the molecular weight, XlogP, the number of H-bond donors and H-bond acceptors.

### Training and validation of the machine learning models

The second step in our model construction pipeline was the choice of ML algorithms suitable for the research question. From the plethora of ML algorithms discussed in the literature with different merits and applicability domains, we adopted and assessed the performance of five different algorithms to answer our research question. These algorithms are:

1. Random Forests (RF)
2. Extreme Gradient Boosting (XGBoost)
3. Support Vector Machines (SVMs)
4. Multi-Layer Perceptron (MLP)
5. Deep Neural Networks (DNN)

The aforementioned algorithms have been successfully applied in previous chemoinformatics and QSAR research studies^33,34^. All selected algorithms exist as integral packages in open-source Python libraries; namely Scikit-learn^35^, XGBoost^36^ and Keras & Tensor-flow^37^. Keras is the high-level Application Programming Interface (API) of Google’s deep learning library Tensor-flow^38^. While Tensor-flow performs all mathematically dense computations, Keras provides a user-friendly, scriptable interface for Tensor-flow, which is excellent for fast prototyping and model experimentation. A brief description of theory behind each of the five used algorithms can be found in the Supplementary Information.

### Technical details

#### Optimization of models’ hyperparameters

Given the complex structure of the dataset and the existence of many tunable hyperparameters in each ML model, it was crucial to perform a crude optimization of those hyperparameters. For each model, we have selected the most important tunable hyperparameters and performed a grid search of those hyperparameters in a fivefold cross-validation protocol for ~ 1% of the dataset. For Scikit-learn based models, we used the “GridSearchCV” method implemented in Scikit-Learn, which creates a unique set of all permutations of the selected space of values of these hyperparameters and determines the best performing set of these hyperparameters’ values. Although not absolutely guaranteed to perform equally well on the full dataset, the method should give some confidence in the quality of the selected hyperparameters.

For the DNN hyperparameters search, the optimization protocol mainly focused on the number of layers. The number of nodes in the input and hidden layers was selected relative to the size of the input features in each category of models (64 nodes for models trained on basic molecular properties; 128 for models trained on the RDkit molecular descriptors and 2048 for a model trained on the ECFP6 fingerprints). The standard “adam” optimizer together with a “categorical cross-entropy” loss function was used for the entire 100 training epochs. Unless otherwise specified, all other hyperparameters were left to the default values. All tunable hyperparameters for all models are given in Table 1. Table 1 also contains the optimum values of the searched space of hyperparameters.

**Table 1:** Selected hyperparameters used for tuning the machine learning algorithms together with the best performing values of these hyperparameters.

ML Models	Tunable hyperparameters	Optimum hyperparameters
RF Model	{"n_estimators":[100,1000,5000],"min_samples_leaf" [3, 5, 10]:}	RF trained on basic molecular descriptors: {"n_estimators":[100],"min_samples_leaf" [10]:} RF trained on RDKit molecular descriptors:{"n_estimators":[5000],"min_samples_leaf" [3]:} RF trained on ECFP6:{"n_estimators":[1000],"min_samples_leaf" [3]:}
XGBoost Model	﻿{"n_estimators":[100, 1000, 5000],"learning_rate":[0.1, 0.01, 0.001], "max_depth" [3, 5, 10]:}	XGBoost trained on basic molecular descriptors: ﻿{"n_estimators":[5000],"learning_rate":[0.001],"max_depth" [3]:} XGBoost trained on RDKit molecular descriptors:﻿{"n_estimators":[5000],"learning_rate":[0.1],"max_depth" [5]:} XGBoost trained on ECFP6:﻿{"n_estimators":[1000],"learning_rate":[0.1],"max_depth" [3]:}
SVM Model	﻿{"kernel":[‘rbf','linear'], "C":[1,10,100,1000]}	SVM trained on basic molecular descriptors: ﻿{"kernel":['rbf'], "C" [10]:} SVM trained on RDKit molecular descriptors:﻿{"kernel":['rbf'], "C" [100]:} SVM trained on ECFP6:﻿{"kernel":['rbf'], "C" [100]:}
MLP model	{"hidden_layer_sizes": [100, 1000, 2000, 5000], "max_iter":[100, 1000, 5000]," alpha":[0.1,0.01,0.001]}	MLP trained on basic molecular descriptors: ﻿{"hidden_layer_sizes":[99], "max_iter":[5000]," alpha":[0.1]} MLP trained on RDKit molecular descriptors:﻿{"hidden_layer_sizes":[1000], "max_iter":[1000]," alpha":[0.1]} MLP trained on ECFP6: {"hidden_layer_sizes":[5000], "max_iter":[1000]," alpha":[0.01]}
DL model	{"Number of hidden layers": [1, 2, 3]}	DL trained on basic molecular descriptors: {"Number of hidden layers": [1]} DL trained on RDKit molecular descriptors: {"Number of hidden layers": [1]} DL trained on ECFP6: {"Number of hidden layers": [1]}

#### Model’s training

All models were fitted on the training set and validated on the test set. As mentioned earlier, the full dataset used for model building and evaluation comprised of 148,128 unique and nonredundant molecules. Of those, 103,689 molecules were used for training and 44,439 molecules were used for validation (70/30 protocol). To mimic the traditional n-fold cross-validation given this large dataset, we initiated the model training and validation in five different and independent runs. In each run, data splitting (training/validation) was randomly made and the models were trained (fitted) and validated from scratch. Data set splitting was performed in a stratified fashion such that an equal proportion of each class of ligands (identified by the corresponding GPCR target) was maintained in the training and the test set. Reported final model evaluations metrics were calculated as the average scores across the five independent runs.

It is often the case that datasets are stored in databases following a specific pattern; such as a temporal or an alphabetical pattern. If this fact was not paid proper attention to, this may result in improper representations of the different classes of observations in the training and the test set. In order to avoid any potential bias in our analysis that may be introduced as a result of the inadequate representations of the chemical space of the input molecules between the training and test set, we randomly shuffled the entire dataframe before being subjected to the dataset splitting (the train_test_split module in Scikit-Learn).

As a further check to confirm the ability of this random shuffling and splitting steps towards achieving a uniform distribution of the input chemical space, the entire dataset (training/test) was projected to lower dimensions using a standard dimensionality reduction technique. In the current study, the t-SNE library from the Scikit-Learn/manifold module was used to map the physicochemical attributes (the 111 RDkit descriptors) of the entire dataset to 3-dimensional vector space and visualized the results in a 3-dimensional-scatter plot.

#### Model validation and assessment

In the current study to facilitate comparison of the predictive power of the different machine learning and deep learning classifiers, several validation scores have been generated for assessing the performance of each model on the validation (test) set. These scores include,

i. $$\mathrm{Accuracy }= \frac{TP+TN}{TP+TN+FP+FN}$$

where *TP* is the number of True Positives, *TN* is the number of True Negatives; *FP* is the number of False Positives and *FN* is the number of False Negatives)

(Accuracy: worst value = 0; best value = 1)

ii. $$\mathrm{F}1\mathrm{ score}=\frac{2\times \mathrm{TP}}{2\times \mathrm{TP}+\mathrm{FP}+\mathrm{FN}}$$

(F1 score: worst value = 0; best value = 1)

As discussed in an excellent review by Chicco^39^, accuracy scores and the F1 scores are somehow misleading as they both can mask the effect of the size of the four classes in the confusion matrix (*TP*: True Positives, *TN*: True Negatives, *FP*: False Positives & *FN*: False Negatives). This is critical when dealing with an imbalanced dataset, i.e. not all classes are equally represented within the dataset, which is the case for most real-world datasets. To solve this issue, the following score has been introduced:

iii. Matthew correlation coefficient scores (MCC)^40^
  $$\mathrm{MCC }= \frac{TP\times TN-FP\times FN}{\sqrt{\left(TP+FP\right)\times \left(TP+FN\right)\times \left(TN+FP\right)\times (TN+FN)}}.$$

MCC score is a robust measure for classification success in case of imbalanced datasets. All validation scores have been generated through the Scikit-Learn library.

### Classification of DrugBank molecule dataset

As a proof-of-concept, our final step was to use the models in real-world applications. As our main focus in the study is to address the problem of drug repurposing, we retrieved the full DrugBank dataset^41,42^ of approved/withdrawn/obsolete/investigational drugs. The dataset was downloaded from (www.drugbank.ca) as a CSV file that contains tabular data for all drugs, ~ 10,255 entries. The dataset was prepared and filtered with the same protocol used for preparing the input data in the model training and validation process (see above); molecular descriptors and fingerprints were generated for each molecule. After preparation and filtering, the final size of the DrugbBank dataset was reduced to 7990 entries, for which prediction using the developed models was performed to evaluate if any of these molecules could have any GPCR binding potential. It is worth mentioning that the final models that we applied on the Drug-Bank database were developed upon training on the full dataset.

### Structure based in silico analysis

We performed an extended analysis by including a structure-based investigation on one of our proposed predictions. We provide here a brief description of the structure-based methods were employed in this study and discuss the purpose and rationale of the investigation in the later section of the manuscript. The crystal structure of the human CB1 was obtained from the Protein Data Bank (PDB ID: 5XR8)^43^. Drugs, including Fenofibrate, Clofibrate, Clinofibrate, and AM841 were selected for the structural analysis. The structures for the ligands were retrieved from ChEMBL database^44^ and consequently prepared using the LigPrep tool in Schrodinger drug discovery suite 2019-3^45^. As reported in the literature, two residues of the CB1 protein, i.e. Asp163, Asp213 are in protonated state when CB1 protein is in its activated state^46^, and accordingly manual protonation was done for these two residues during the modeling process. Molecular docking simulations of the ligand structures to the CB1 protein were performed using MOE2019 software^47^ through the induced fit algorithm. The docking grid was set up using the binding site residues reported in the literature^43,48^. This led to 150 poses for each ligand, which were further refined to 50 poses and ranked by RMSD fit analysis. The latter revealed that the ligands possessed on average four main orientations in the binding site and thus four poses for each ligand were finally selected adding up to thirteen protein–ligand complexes (see below).

Next, using CHARMM-GUI Membrane Builder^49^, the complexes were embedded in a lipid bilayer composed of Palmitoyloleoylphosphatidylcholine (POPC) lipid molecules. The lipid bilayer was further solvated with TIP3 water molecules and ions (NaCl conc. 0.15 mM). MD simulations were performed using NAMD package with CHARMM36m forcefield^50^. The simulation protocol was adapted from our earlier publications^51,52^. The production run added up to 10 ns, which was subsequently sampled every 4 ps for binding free energy calculations using MMGBSA algorithm in AMBER14^53^.

### Python libraries versions

All models were generated using open-source Python libraries. We used Python 2.7, Miniconda2 distribution. Initial data exploration was performed on a MAC Pro; final data preparation and model generation steps were performed on CEDAR, a linux-based high performance computing cluster from Compute Canada. We used the following packages: Pandas (0.22.0), DeepChem (2.1.0), Numpy(1.15.2), Scikit-Learn(0.20.0), XGBoost(0.6a2), Keras(2.2.4) & Tensor-flow(1.3).

## Results and discussion

The current study encoded all known GPCR ligands listed in the GLASS database through their molecular descriptors (i.e. physicochemical properties) and their extended-connectivity fingerprints (ECFPs) into ML models. The ultimate goal of these models is to aid in the process of identifying novel drug-repurposing opportunities for the GPCR targets. Additionally, these models can be also used in general drug screening campaigns against GPCRs. To construct these models, we first generated unique sets of features of the input ligands. Towards this goal, we utilized both the molecular descriptors and the molecular fingerprint representation methods. Using these molecular representations, we developed 15 supervised ML models encoded in open-source Python libraries. The evaluation of performances of these models show that the DNN models achieved the highest classification power with an MCC score of ~ 92.2%, slightly better than other algorithms (see below). We then used these models in a practical scenario and screened the Drug-Bank database of FDA approved and investigational drugs to identify potential hits against the GPCR receptors. Using our ECFP6-based models in a consensus-based fashion, we identified potential interactions beyond what was already listed in the GLASS database. We then validated our findings through experimental data found in the literature and confirmed these interactions through structural modelling. Below is a detailed discussion of our findings and also a discussion of the limitations of the current study for a future perspective.

### Assessing the uniform distribution of the chemical space using t-SNE

It is important to visualize the full dataset in a low dimensional representation to ensure the proper chemical space overlap between the training and test set. Traditional linear dimensionality reduction methods, such as Principal Component Analysis (PCA) can be used for data visualization only if the first 2 ~ 3 dimensions (principal components) are able to capture a large proportion of the embedded variance within the dataset, which was not the case in our dataset (see below). PCA is an orthogonal transformation where the number of generated dimensions (principal components) is equal to the input features and sorted by the explained variance. On the contrary from linear methods for dimensionality reduction, non-linear methods, such as t-distributed Stochastic Neighbor Embedding (t-SNE) has the capacity to efficiently map the extremely high dimensional space dataset into a few dimensions suitable for data visualization^54^. Furthermore, t-SNE is able to preserve both the local and global structure of the processed observations.

Initially, we tried PCA and the first three eigenvectors (principal components) explained only ~ 50% of the variance within the dataset (data not shown). To create a proper visualization using PCA, one should not exceed 2 ~ 3 principal components in order to adequately explain the majority of variance within the dataset. This indicates that PCA was not suitable to reduce the dimensions of our problem. Therefore, we decided to use the t-SNE, a relatively recent and robust machine learning dimensionality reduction technique with many successful applications in health research, particularly in genomics^55^, subcellular localization^56^ and human diseases^57^. We used the extensive set of RDkit generated molecular descriptors (111 descriptors) as an input for the t-SNE analysis. The full dataset composed of ~ 148,000 molecules.

**Figure 1:**
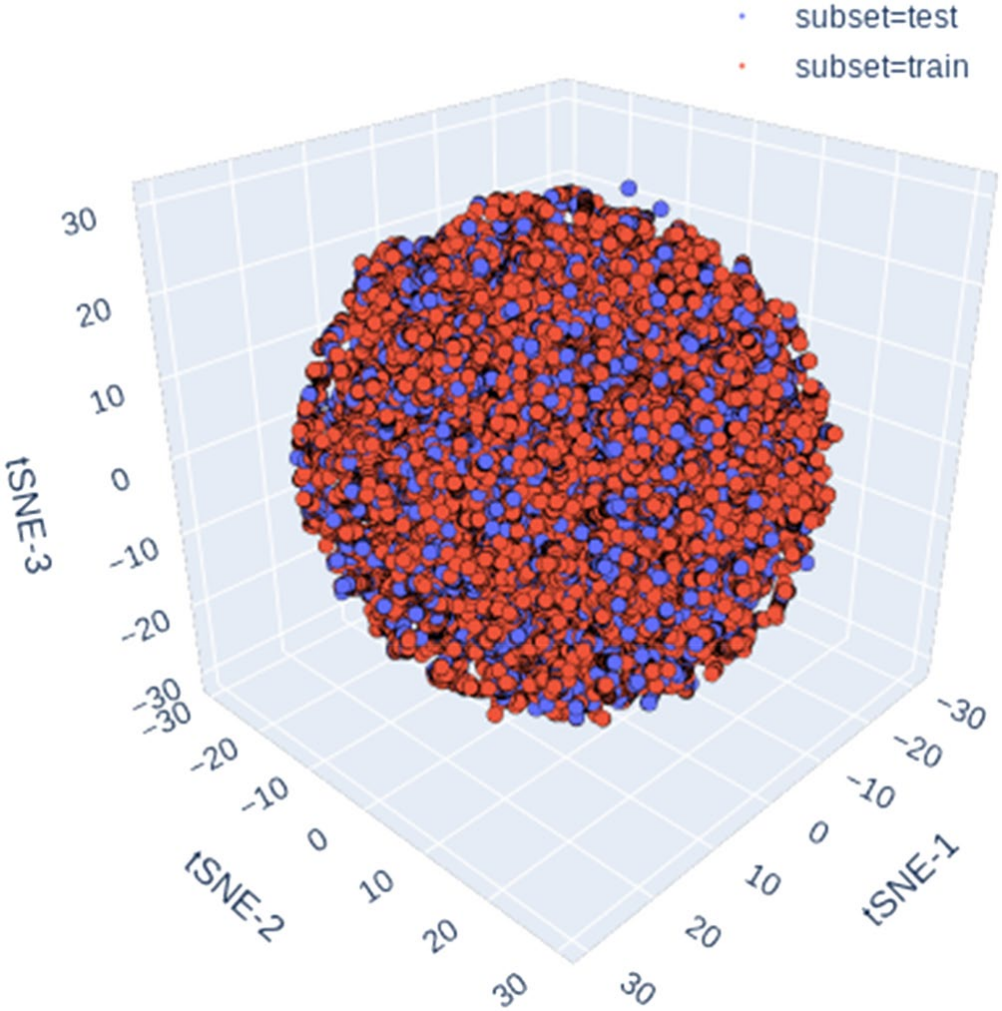
A 3D scatter plot of the three t-SNE axes projected over the RDKit-descriptors space. Data points are colored according to the belonginess of the data point to either the training or test dataset. The figure was generated with Python Matplotlib 3.2.1: https://matplotlib.org/.

Figure 1 shows the 3D scatter plot of the mapped t-SNE vector space. Data points are colored by their classification to either the training or to the test sets (see legend). As expected, the uniform, scattered distribution of the mapped t-SNE vector spaces reflects the inherent structural heterogeneity of the input dataset that covers a broad range of continuous features values. Furthermore, it is clear from the homogenous overlap between the training and test sets’ scattered data points that both datasets cover the entire feature space of the input molecules.

### An overview of the physicochemical properties of the selected compounds dataset

**Figure 2:**
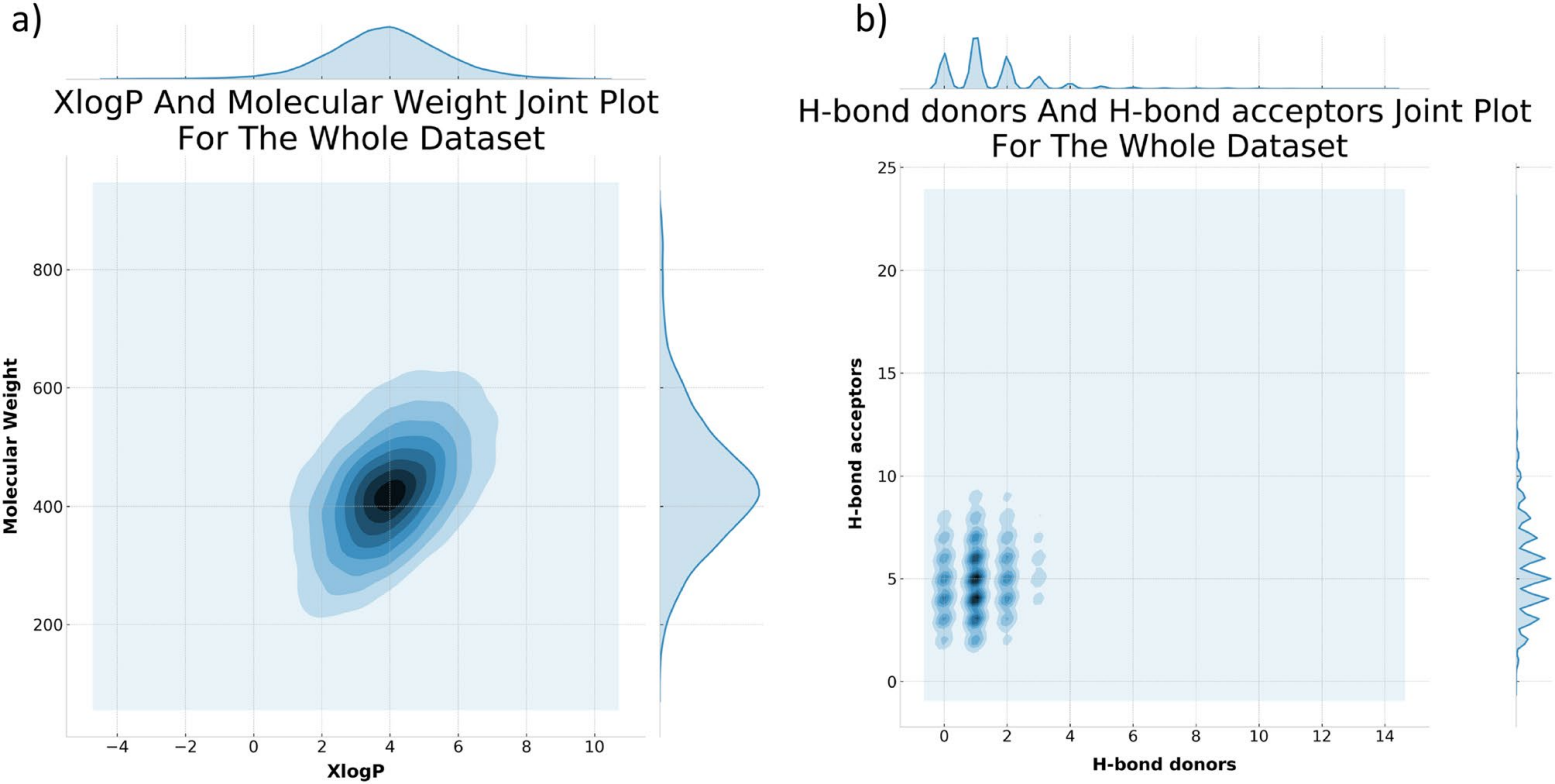
Two joint plots showing the distribution of the selected range of physicochemical properties considered in the current study, (**a**) Molecular weight & XlogP, and (**b**) H-bond acceptors & H-bond donors. The figure was generated with Python Matplotlib 3.2.1: https://matplotlib.org/.

The four most important physicochemical properties of drug-like molecules are their molecular weights, molecular lipophilicity, number of H-bond donors and number of H-bond acceptors. Figure 2 shows two joint plots of these properties for all the compounds included in our analysis. As shown in Fig. 2a, the vast majority of the compounds are centered around a molecular weight of ~ 400 Da, and an XlogP of ~ 4. For the H-bond donors and acceptors (Fig. 2b), the majority of compounds have ≤ 2 H-bond donors, and 3:8 H-bond acceptors. The data coincides with the fact that the dataset is filtered to the normal range of drug-like organic compounds commonly employed in drug screening studies. The filters, however, are not very strict given the fact that many marketed drugs violate the recommended Lipinski range of drug-like molecules^31,58^.

**Figure 3:**
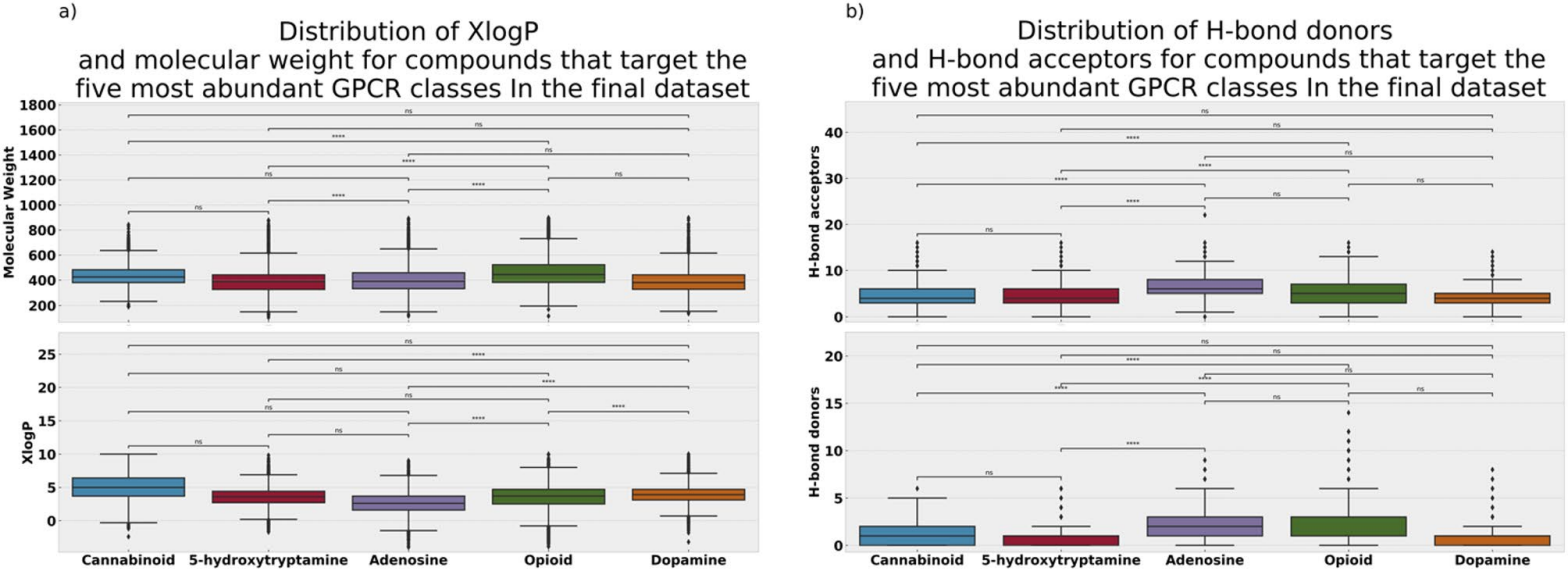
Two box-plots displaying the physicochemical properties distributions of the five most abundant GPCR targets represented within the selected subset of the GLASS database; (**a**) Molecular weight & XlogP, and (**b**) H-bond acceptors & H-bond donors. Statistical tests of significance were calculated using the statannot 0.2.3 python library, through the Mann–Whitney-Wilcoxin test, with Bonferroni correction: ns: 5.00e−02 < p ≤ 1.00e + 00. *: 1.00e−02 < p ≤ 5.00e−02. **: 1.00e−03 < p ≤ 1.00e−02. ***: 1.00e−04 < p ≤ 1.00e−03. ****: p ≤ 1.00e−04. The figure was generated with Python Matplotlib 3.2.1: https://matplotlib.org/.

To visualize the class-specific distribution of the aforementioned physicochemical properties, we generated the box-plots (see Fig. 3) of these properties for the five most abundant target classes in the final dataset. These classes include 5-Hydroxytryptamine, Cannabinoid, Adenosine, Opioid, and Dopamine. With the exception of Adenosine, the rest of the most abundant GPCR targets are Central Nervous System (CNS) specific targets. As shown in Fig. 3a,b, the compounds possess a distinctive range of values across the set of selected physicochemical properties. On average and as shown in Fig. 3a, the Cannabinoid class of compounds is the most lipophilic (median XlogP ~ 5.0), possessing the second highest median molecular weight value (~ 424.5 Da) following the opioid targeting compounds (~ 443.6 Da). Of note are the opioid receptor ligands that exhibit a relatively low median XlogP of 3.7, which may be attributed to the presence of permanently charged basic center in many of their derivatives^59^. Being a non-CNS exclusive target, the adenosine targeting compounds possess the lowest XlogP overall (median XlogP ~ 2.6), presumably due to the lack of need for blood–brain barrier penetration.

For the H-bond acceptors (Fig. 3b, lower panel), the highest number of H-bond acceptors is attributed to Adenosine, the non-CNS exclusive target (median H-bond acceptors ~ 6). For the H-bond donors (Fig. 3b, upper panel), Adenosine targeting compounds retain the highest number of H-bond donors (median H-bond donors ~ 2), similar to opioids targeting compounds.

### GPCRs targets and molecular diversity

In order to evaluate the likelihood of the investigated GPCRs to bind chemically diverse ligands, each class of ligands were clustered using a Tanimoto similarity index of 0.8, using the RDKit implementation of the Butina clustering algorithm^60^ in RDKit. Ligands were encoded through the ECFP6, radius 4 fingerprints. As the number of clusters are expected to be artificially inflated by the input size, a size-calibrated diversity index was calculated for each class of ligands (diversity index = Total number of clusters/Total number of ligands), such that a high diversity index indicates a ligand class with greater diversity. The analysis was performed for the entire list of GPCRs. However, we only focus here on those GPCRs with at least 5000 interaction records available in the used dataset. The full results is provided as an excel sheet in the supplementary materials (see Supplementary S2). In brief, our findings suggest that the Dopamine receptors bind the most diverse ligands with a diversity index (Total number of clusters/Total number of ligands) of 0.058 followed by 5-hydroxytryptamine receptor with a diversity index of 0.043 and Histamine with a diversity index that of 0.039. On the other hand, the CC chemokine receptors was shown to bind the least diverse ligands, achieving a diversity index of 0.027. These results should be interpreted in the context of therapeutic relevance as there will be always more diverse molecular scaffolds developed for pharmaceutically important GPCR drug targets.

**Figure 4:**
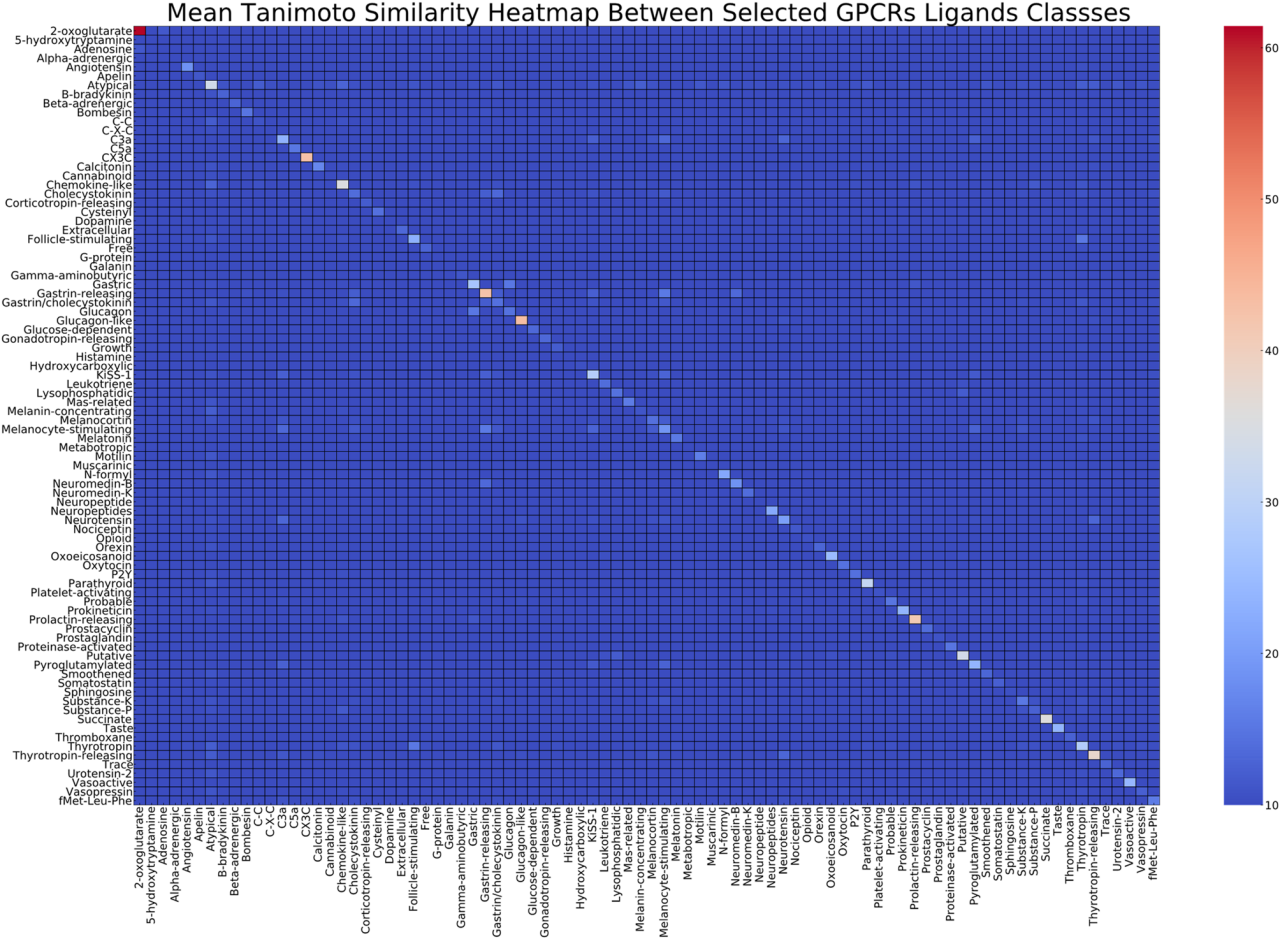
Mean Tanimoto similarity heatmap of the studied classes of GPCRs ligands. For the similarity analysis, ligands are represented through the ECFP6, radius 4 fingerprints.

To investigate the inter-classes ligands similarities, we have calculated the average Tanimoto similarities between each pair of GPCRs ligands. Generated square matrix was provided as a heatmap (Fig. 4). The diagonal of the matrix should evaluate the intra-class ligands similarity, i.e. ‘ligands homogeneity’. As shown in Fig. 4, As expected, matrix’s diagonal that showed the average intra-class ligand similarity produced the highest similarity overall, with maximum intra-class homogeneity achieved by 2-oxoglutarate ligands (mean Tanimoto similarity of 61.4%), and a minimum value is achieved by Metabotropic receptor (mean Tanimoto similarity of 9.0%). Although the analysis should not be studied in isolation from the therapeutic importance of the targets as we have illustrated in the previous section, certain interesting patterns can be identified. For example, the Gamma-aminobutyric ligands shows the highest dissimilarity from other classes of GPCRs targets. Also, the somehow related GPCR target classes, Gastric and Glucagon receptors exhibited a mean Tanimoto similarity of 15.2%. The raw numbers were appended to the S2 excel file as a separate spreadsheet.

### Developing supervised ML models and evaluating the classification performance on the test set

The previous sections discussed the construction of the datasets used for model training and validation. Initial models’ hyperparameters were optimized in a fivefold cross-validation experiment on a subset of the input dataset. This optimization was necessary to select the best combination of hyperparameters for the Scikit-learn GridSearchCV algorithm (for the ML models) and to tune the number of hidden layers in the DNN models.

**Figure 5:**
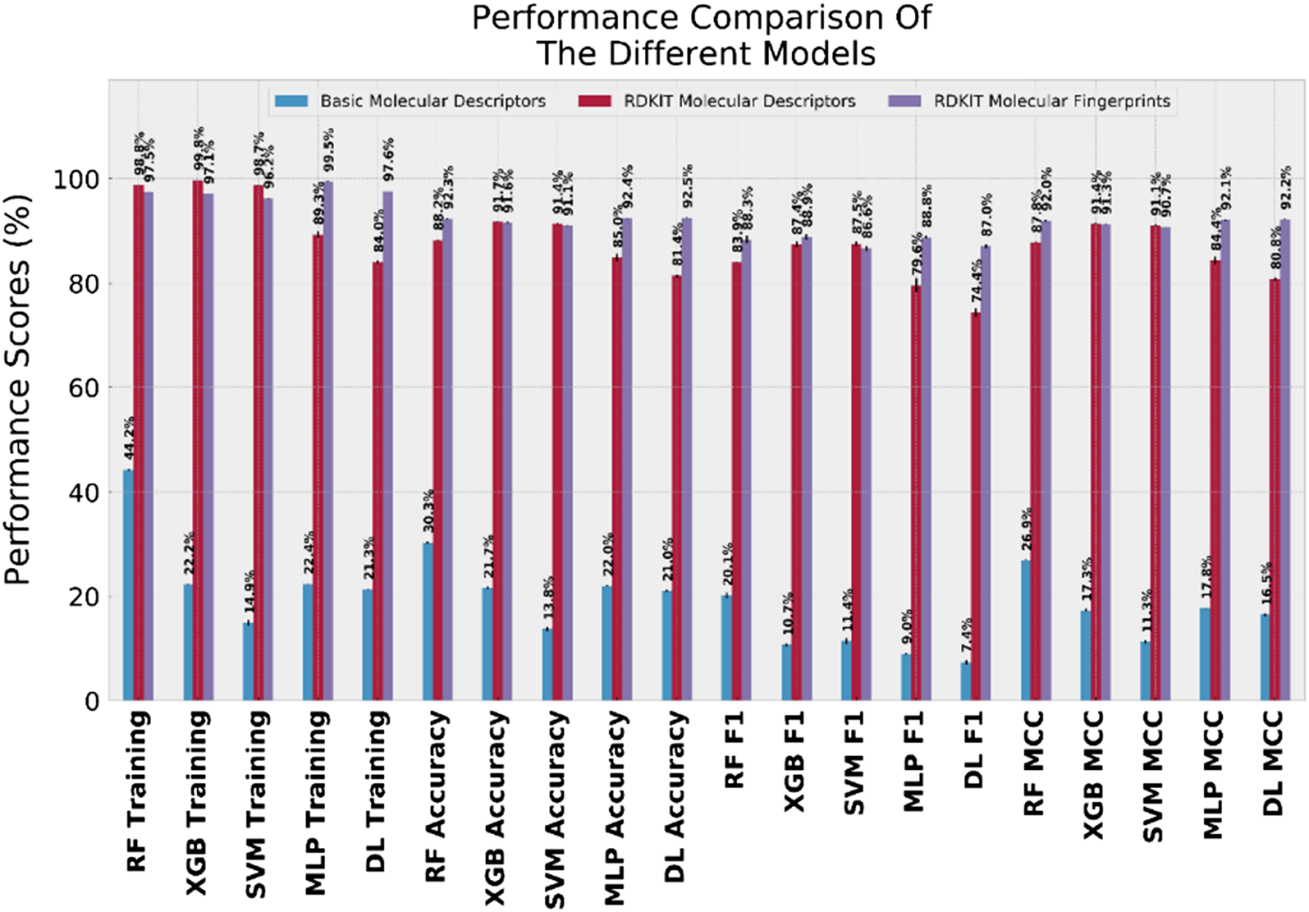
Performance scores of the 5 different machine learning models considered in the current study. Reported scores represent the average performance of the 5 repetitions on the independent test dataset. In each repetition, data is split according to the 70/30 protocol. The figure was generated with Python Matplotlib 3.2.1: https://matplotlib.org/.

Below, we will assess the predictive performance of the trained models using a test dataset. It is worth mentioning that the reported scores here are the mean values of the collected scores from five different and independent model training/validation runs. The final model scores were obtained by testing the predictive power of the models on a test dataset, which was not included in the training process of the models in the corresponding run. As a result of the inherited imbalance in the dataset used for training and validation, it was important to calculate the performance scores that can overcome this imbalance limitation. Consequently, common model evaluation metrics such as accuracy, precision and recall are not be suitable for such cases. Therefore, in addition to accuracy, we have also reported more dataset imbalance robust metrics, including F1-scores (the harmonic mean of precision and recall) and Mathew’s correlation coefficient (MCC) scores. The performance scores of all models are illustrated as a bar chart in Fig. 5. Given the reported superior assessment power of the Mathew correlation coefficient scores for imbalanced dataset classification algorithms, we limit our discussion here to this score. Nevertheless, similar conclusions can be made from other scores as well.

Regardless of the values of the training scores obtained by evaluating the generated models on the subset of data used for training, the true measures to assess the model’s performance are usually obtained by assessing the predictive power of the models on the subset of data kept aside for validation (i.e. the validation test) scores. In supervised machine learning, high training scores and low validation scores most probably mean that the model has been overfitted on the training data such that its generalization performance is weak (low bias, high variance problem)^61–63^. On the other extreme, a model that has weak training and validation scores is usually described as being an under-fitted model (high bias, low variance problem). Generating an overfitted or an under-fitted model greatly diminishes the usefulness of the modeling process. A good model will exist in the sweet spot; it has both a good training score and a good validation score.

As shown in Fig. 5, regardless of the modeling algorithm and the selected scores, a higher number of input features usually lead to better predictive performances. Not surprisingly, models that are trained on the RDkit generated molecular descriptors (111 molecular descriptors) or on the ECFP6 fingerprints performed substantially better than those trained on the four basic molecular descriptors retrieved from GLASS (Molecular Weight, XlogP, HBD/HBA). Furthermore, molecular fingerprints-based models are shown to perform generally better compared to models trained on the RDKIT generated molecular descriptors. For example, the MCC score of the RF model trained on the RDKIT-descriptors is given by 87.8 ± 0.11%, which is lower than the corresponding score of the same model trained on the ECFP6 (i.e. 92.0 ± 0.15%). The same trend is observed for all models except for the SVM & XGB models, where both representation methods performed similarly (i.e. XGP: 91.4 ± 0.13% and 91.3 ± 0.1%; SVM: 91.1 ± 0.13% and 90.7 ± 0.07% for models trained on RDKIT-descriptors and ECFP6, respectively). The trend is particularly obvious in Artificial Neural Networks-based models (MLP and DNN). Of note, the Deep-ANN model performed best overall with an MCC score of 92.2 ± 0.07%. The high training scores suggests that there are still a room for improvement, which will be attempted in future subsequent studies. An excel (.xlsx) file that contains all training and validation scores is included in the Supplementary Information S3.

### A practical application of the models

Given the excellent predictive performance of the developed models on classifying the training as well as the test datasets, it was encouraging to apply these models in real-world scenarios. The prepared Drug-Bank dataset was used as an input for the models to predict potential GPCR target classes. Given the superior performance of models trained on ECFP6, we limited our analysis for prediction entries where all ECFP6-based models (RF, XGB, SVM, MLP, and DNN) show consensus for the predicted GPCR target classes. Consensus was achieved for 1802 unique ligands out of 7990 DrugBank ligands screened (22.6%). This is close to the latest estimate we can find in the literature for GPCRs targeting drugs (∼ 25 to ∼ 36%)^26^.

Next, we examined the prediction output to explore potential interactions that can be highlighted. As we were aware there could be potential overlap between GLASS and the DrugBank dataset, we split the prediction output into two different categories based on the presence of a particular interaction in GLASS. The full data is provided in a single Supplementary excel file S4 with three spreadsheets, (1) Full predictions output (1802 records), (2) predictions that are not within the exploited subset of GLASS (1299 records), and (3) predictions that are already listed within the exploited subset of GLASS (503 records). In the following section, we will discuss few examples for interactions that were not listed in the exploited subset of GLASS. Later, we will briefly mention about few examples of common interactions that already listed in GLASS and what can we learn from these examples.

### Showcases of potential success

**Figure 6:**
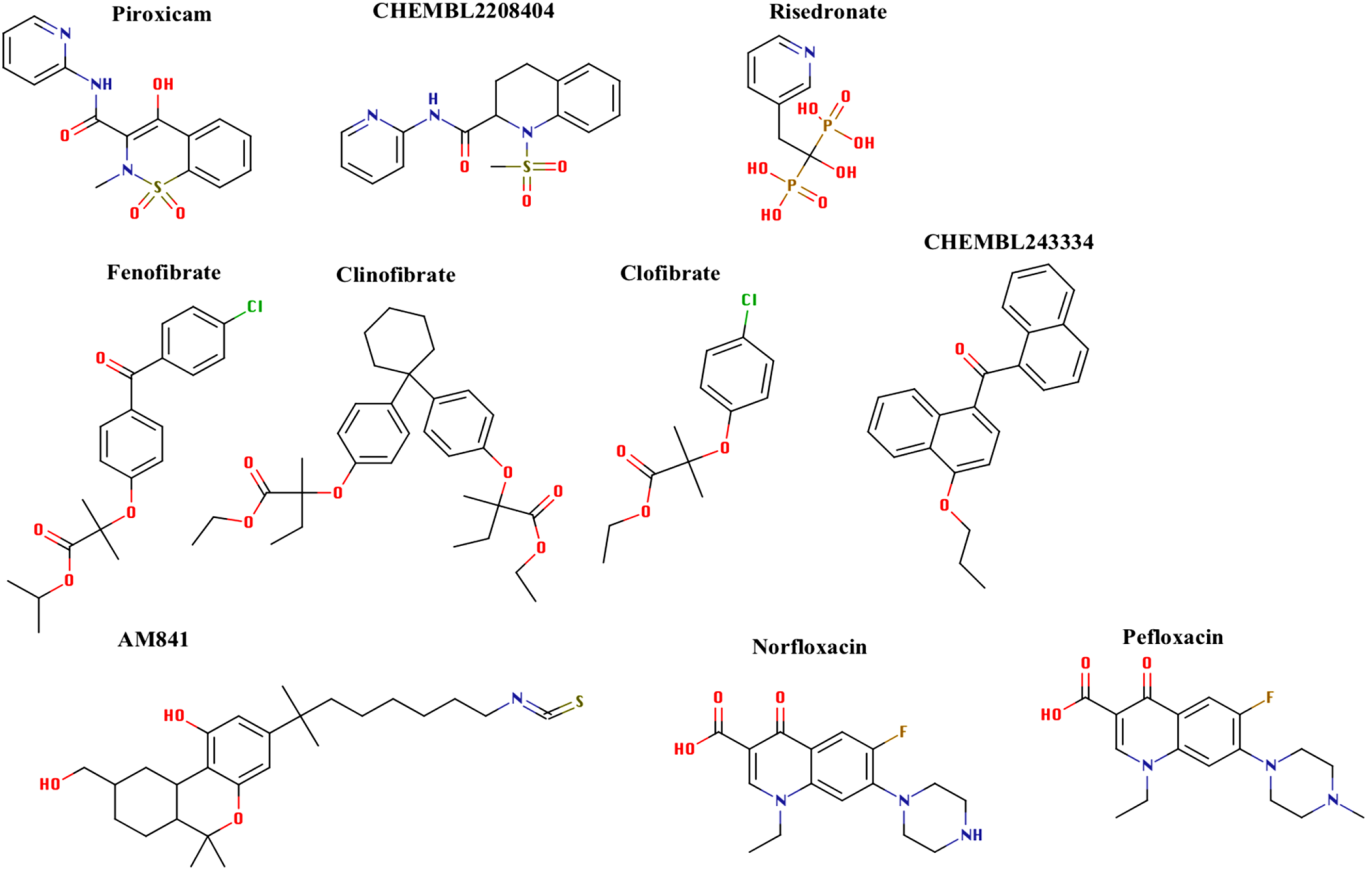
The chemical structures of the compounds predicted as potential ligands for GPCRs; Piroxicam & Risedronate (potential Glutamate receptor ligands), Fenofibrate, Clinofibrate & Clofibrate (Potential Cannabinoid receptor ligands), Norfloxacin & Pefloxacin (Potential 5-Hydroxytryptamine receptor ligands). The figure also includes the chemical structures of CHEMBL2208404: a glutamate receptor agonist listed in GLASS and is structurally similar to Piroxicam; CHEMBL243334: a cannabinoid receptor agonist listed in GLASS and is structurally similar to Fenofibrate; AM841 (a co-crystallized cannabinoid agonist). The figure was generated with MarvinSketch: https://chemaxon.com/products/marvin.

Here, we show three different examples for using the developed models in a practical drug repurposing scenario. The first example reveals the ability of the popular non-steroidal anti-inflammatory drug (NSAID), Piroxicam (Feldene), to interact with the metabotropic glutamate receptors. Piroxicam (see Fig. 6) is an enolic oxicam derivative and is well known for its long-lasting effect^64^. In previous studies, certain members of the NSAIDs family, including Piroxicam, have been shown to interfere with the ionotropic *N*-methyl-d-aspartate (NMDA) receptors. These interactions, however, remained ambiguous. Piroxicam was shown to have a beneficial effect in certain forms of brain ischemia, which is closely linked to the level of glutamate^65^. It is known that the ionotropic glutamate NMDA ion channels are tightly regulated by the metabotropic glutamate receptors, which is a GPCR^66–69^. Based on our model’s prediction, we propose that piroxicam exerts its NMDA effect through modulation of the activities of the metabotropic glutamate receptors, which in turn regulates the activities of the ionotropic glutamate NMDA ion channels. The closest piroxicam structural analogue that is also listed as a metabotropic glutamate receptor ligand in GLASS (CHEMBL2208404) possesses approximately 60% Tanimoto similarity with piroxicam.

Our second example is risedronate, a bisphosphonate drug that is commonly used in osteoporosis. Our models predicted that risedronate (see Fig. 6) could potentially interact with the metabotropic glutamate receptors. This is in accordance with recent data from a study by Shima et al., which showed that bisphosphonates could exert their potential analgesic activities through interaction with the Glutamate pathway^70^. None of the bisphosphonate ligands or their structural analogues are listed in the GLASS database.

As a third example, our models suggest that the popular fluoroquinolones antibacterial agents (Norfloxacin & Pefloxacin) can interact with the 5-hydroxytryptamine (5HT) receptors, a family of CNS receptors that are majorly involved in controlling aggression, anxiety, appetite, cognition, learning, memory, mood, nausea, sleep, and thermoregulation^71^. Given the superior activities of the fluoroquinolones antibacterial agents in many serious bacterial diseases, this observation was interesting to investigate. There are many reports concerned with the involvement of fluoroquinolones in many CNS related complications^72,73^. This includes peripheral neuropathy, hallucinations, anxiety, depression, insomnia, severe headaches, and confusion. A systematic search for CNS related complication was conducted by Tandan et al. who showed that the risk of developing CNS related complications with fluoroquinolones are ~ 3 times higher than with any other antimicrobial agent^74^. Furthermore, recent case studies regarding another fluoroquinolone, Moxifloxacin, showed that the drug is closely associated with the development of visual hallucinations, alterations in mood and behavior, and some severe forms of acute psychosis^75,76^. It is obvious that many of these CNS complications overlap with the physiological roles of the 5HT receptors. In the GLASS database, norfloxacin is listed as an agonist for other receptors, not including 5HT, and none of the GLASS listed structure analogues, up to 70% Tanimoto similarity threshold, was listed as potential 5HT agonists. Whether flouroquinolones achieves a direct interaction with 5HT receptors is an observation that deserves further experimental investigation.

In three examples presented above, although there have been some proofs for potential interactions, additional studies are warranted to clearly prove the direct binding/interactions. One should also keep in mind that there is still a possibility that the nature of the interaction in a given receptor implies an indirect/allosteric interaction, which is a completely different research question per se. Below, we will select one predicted interaction where a direct, unambiguous, competitive binding was experimentally verified. Our focus on this selected example is to investigate the nature of such interactions at the finest possible atomic level of details.

### A complete case study: computationally identified approved drug for cannabinoids receptors (Clinofibrate and Fenofibrate)

Our ML prediction pipeline identified Clinofibrate and Fenofibrate as potential ligands for the Cannabinoid receptors. In the GLASS database, fenofibrate is listed as a potential ligand for a few GPCRs, not including Cannabinoid receptors, while Clinofibrate is not listed. We searched GLASS for Cannabinoid receptor agonists that are structurally similar to Fenofibrate, using a Tanimoto similarity threshold of 50%. Interestingly, we found that the closest Cannabinoid receptor agonist is CHEMBL243334 (see Fig. 6), which shares ~ 61% Tanimoto similarity with Fenofibrate. Furthermore, studying this potential interaction at the atomistic level of details leads us to a few interesting conclusions (see below). It is noteworthy to mention that previous reports showed that certain cannabinoids can modulate the activity of PPARs receptors^77^, a phenomenon that may explain why our models predicted a well-known PPARs agonist as a potential cannabinoid receptor agonist. These earlier reports suggested that these effects could be due to involvement of downstream signaling cascades^77^. Based on the results obtained from our model, we are proposing that a straightforward poly-pharmacological effect of these cannabinoids, through direct binding, could be a better explanation.

Our structural analysis involves docking and MD simulation of fenofibrate, clinofibrate, clofibrate and AM841 (a co-crystallized cannabinoid agonist) against the well-known cannabinoid binding site within the CB1 receptor. Fibrates were modeled in the ester form. Please note that fibrate derivatives (i.e. Fenofibrate and Clofibrate) are represented in their ester forms in the GLASS database, whereas the free carboxylate form is the one that was reported in the Drug-Bank database. Clinofibrate (modelled in the ester form here) was not listed in GLASS.

### Predicted structural interactions and estimated AMBER/MM-GBSA binding energies

As discussed above, our models identified some interesting connections between the known drugs from Drug-Bank and members of the GPCR family of proteins. One of these predictions that captured our attention was the activity of fenofibrate and clinofibrate against cannabinoid receptors. Upon a closer look into the available experimental evidence in the literature, we came across a number of experimental studies that had previously investigated the role of fibrates and more specifically fenofibrate as a potential agonist for cannabinoid receptors^78–80^. This strong experimental proof urged us to perform a structural study to test the hypothesis of potential agonistic activity of fenofibrate and clinofibrate against the cannabinoid GPCRs. Cannabinoid receptors belong to the class A GPCR family, comprising two main members, namely the cannabinoid receptor 1 (CB1) and cannabinoid receptor 2 (CB2)^81^. CB1 is expressed throughout the body, with the highest levels found in the central nervous system and mediates the functional responses to the endocannabinoids and the widely consumed plant-driven phytocannabinoid Δ^9^-tetrahydrocannabinol (THC)^82,83^. CB1 has been the target of numerous drug discovery investigations and acts as a potential therapeutic target for the management of pain^84^, inflammation^85^, obesity^86^, epilepsy^87^ and many more emerging medical applications^88,89^.

To shed more light on the primary predictions of our model, we selected three members of the fibrates’ family of medications, namely fenofibrate and clinofibrate (predicted to be active against the cannabinoid receptor by the ML model) and also clofibrate which was not predicted by the model to be involved in any Cannabinoid binding activity and was used as our negative control in this study. Fibrates are a widely used class of lipid-modifying agents effective in managing hypertriglyceridemia and hypercholesterolemia through activation of specific transcription factors belonging to the nuclear hormone receptor superfamily, termed peroxisome proliferator-activated receptors (PPARs)^90^. To test the ligands, we used the crystal structure of the human CB1 receptor (PDB ID: 5XR8) as a representative target structure^43^. This structure is co-crystallized with AM841, a hexahydrocannabinol, which is known as a potent full agonist of CB1^91^. Therefore, this structural analysis study included four ligands in total, namely fenofibrate, clinofibrate, AM841 (as our positive control) and clofibrate (as our negative control) (see Fig. 6).

The binding free energy calculations revealed the affinity of each of the ligands towards the CB1 protein (see Table 2). Interestingly, our results show that fenofibrate and clinofibrate bind to the CB1 protein with an average MMGBSA free binding energy of − 37.58 ± 2.7 kcal/mol and − 66.48 ± 4.98 kcal/mol, respectively, which is close enough to the that of the potent CB1 agonist, AM841, which showed a binding energy of − 63.50 ± 4.98 kcal/mol. Clofibrate on the other hand has a much weaker predicted binding energy of on average − 26.86 ± 0.9 kcal/mol indicating its less relevance to CB1 agonism. To reveal more insights on these interactions, further analysis was performed to understand the binding mode of these ligands within the CB1 receptor. Of note, lipophilic residues dominate the binding pocket, which can explain the reason behind the ester form of the drugs being the active form on Cannabinoid receptors. It is also important to mention that we considered multiple poses (the 4 best poses of each ligand) as suggested from docking in our estimation of the binding affinities. Below is a description of the most probable binding modes that were identified for each compound. We focused our structural analysis on the best binding mode as suggested by the MMGBSA estimation of the binding affinities.

**Table 2:** Binding Free Energy Results for the four ligands (AM841, Fenofibrate, Clinofibrate and Clofibrate). The protein–ligand complexes are numbered sequentially from 1 to 4 for the fibrate ligands referring to four independent poses obtained from docking.

Drug–protein complex	Average MMGBSA binding free energy (kcal/mol)	Average binding free energy (kcal/mol)
CB1-AM841	− 63.50	− 63.50 ± 3.5
CB1-Fenofibrate_1	− 38.64	− 37.58 ± 2.7
CB1-Fenofibrate_2	− 39.77	
CB1-Fenofibrate_3	− 33.64	
CB1-Fenofibrate_4	− 38.28	
CB1-Clinofibrate_1	− 61.75	− 66.48 ± 4.98
CB1-Clinofibrate_2	− 66.00	
CB1-Clinofibrate_3	− 73.46	
CB1-Clinofibrate_4	− 64.69	
CB1-Clofibrate_1	− 27.21	− 26.86 ± 0.9
CB1-Clofibrate_2	− 27.89	
CB1-Clofibrate_3	− 25.80	
CB1-Clofibrate_4	− 26.55	

**Figure 7:**
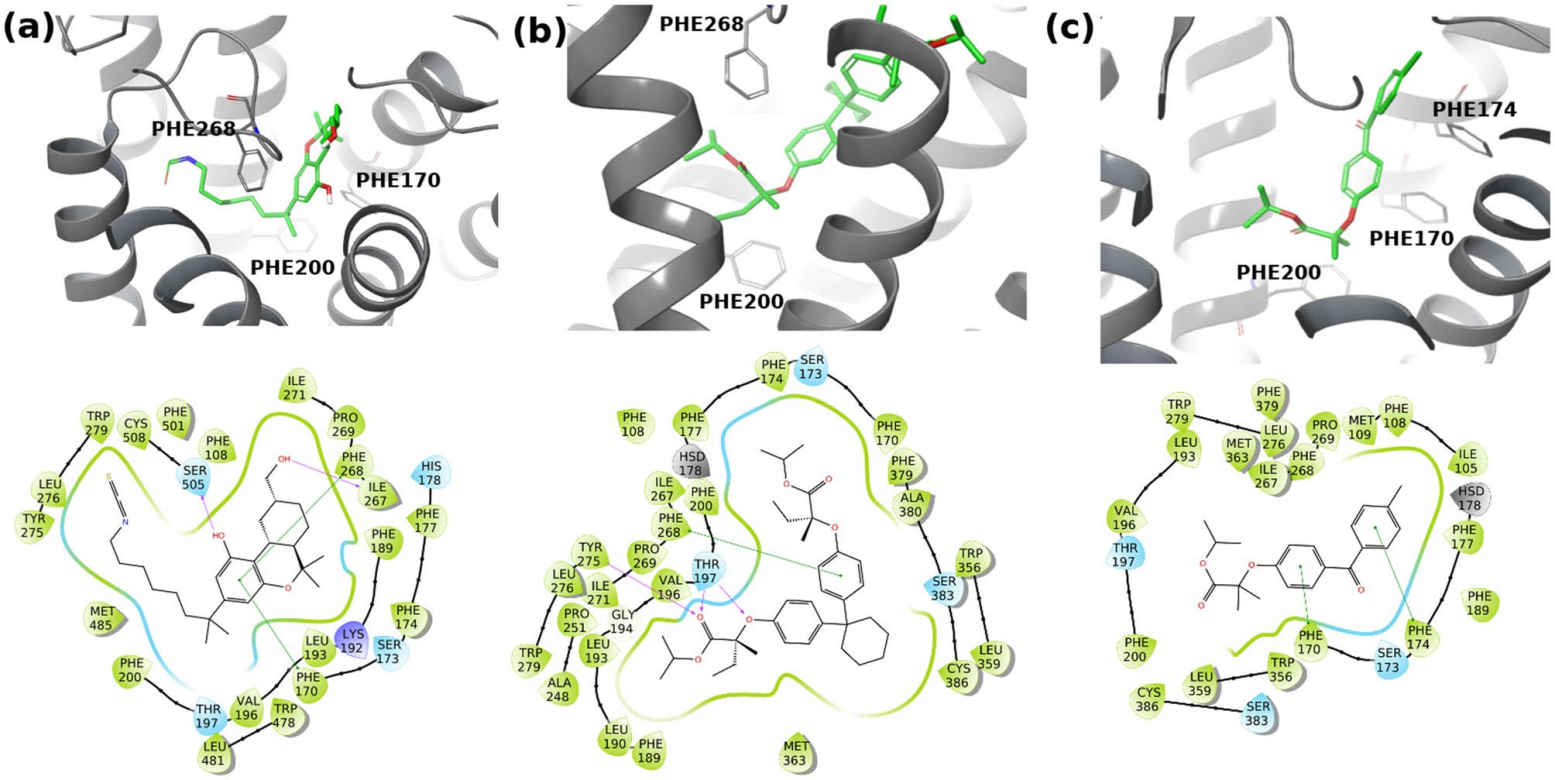
The 3D (upper panel) and 2D (lower panel) ligand interaction diagram of (**a**) the crystal structure of AM841 bound to the Cannabinoid receptor (CB1), (**b**) the potential binding mode of Clinofibrate bound to CB1; and (**c**) the potential binding mode of Fenofibrate bound to CB1. Figure 6 was generated with Maestro 9.2, the Schrodinger suite: https://www.schrodinger.com/products/maestro.

As shown in Fig. 7a, AM841 adopts an L-shaped binding mode where the hexahydrobenzo-[c]chromen ring faces the N-termial of the CB1 receptors and is surrounded by a cluster of PHE residues, including PHE170, PHE174, PHE177, PHE189, PHE268 and PHE379. The lipophilic aliphatic octyl side tail is, similarly, surrounded by lipophilic residues, including PHE200, LEU193, LEU276, LEU359 and MET363. As clinofibrate is a symmetric drug molecule, an α-like binding mode was noticed. As depicted in Fig. 7b, the isopropyl ester group superposes very well with the aliphatic dimethyl moiety of AM841. Being a bulky molecule, clinofibrate completely filled the binding site, forming almost all the interactions that were observed for AM841 within the crystal structure. Fenofibrate (see Fig. 7c) showed a less filling to the binding site, even though the majority of residues’ interactions are preserved. The small molecular size of clofibrate can partly explain its weaker binding affinity towards CB1. Although clinofibrate and fenofibrate were predicted as CB1 ligands by our ML models and also showed strong binding to CB1 according to the MMGBSA binding affinity caclaution, cliofibrate was not predicted to be a CB1 ligand and it also showed a weak estimated MMGBSA binding affinities.

Furthermore, using RDkit, we performed a maximum common substructure search analysis between CHEMBL243334, fenofibrate, and clinofibrate. The three compounds (shown in Fig. 6) share a common biphenyl-ethoxyl motif that could be the reason behind their activities/potential activities against Cannabinoid receptors. Further investigation is currently in progress to shed more light on this observation.

It is noteworthy to mention that our ultimate goal for including the atomistic simulation was to shed light on the rationale underneath this prediction (Fenofibrate and Clinofibrate), and why neither an experimental observation nor a similar ML prediction could be established for Clofibrate, a closely related analog. Our atomistic simulation data provided a structural rationale for these predictions. It was shown that whereas Fenofibrate and Clinofibrate were able to engage with critical residues at the binding site, Clofibrate did not achieve the same level of surface contacts.

In the previous examples, the predictions were not listed in GLASS, which illustrates the usefulness of our models in a real-world scenario. However, we were also interested to analyze the full DrugBank database, including ligands’ interactions that were already listed in GLASS to test if our models can identify these interactions. Our analysis revealed that the models performed well in predicting the correct GPCR target whether the target was listed either as a primary or as a secondary target for the particular compound. As an example of ligands that have a GPCR as their primary target are the known synthetic atropine structural analogues (e.g. Ipratropium). This class of compounds was predicted to interact with the muscarinic receptors. It is worth noting that our model was not trained to differentiate between agonists and antagonists, however, it was trained to evaluate if a compound can interact or not with the target. Another example is the well-known family of Angiotensin-II receptor blockers family of antihypertensive drugs (Sartans, e.g. Losartan and Eprosartan), which were predicted to bind the Angiotensin receptors. As examples of ligands where the secondary target is a GPCR, many of the diazepam family of tranquilizers (e.g. Alprazolam and Triazolam) were predicted to interact with Platelet-activating factor receptor, which is a GPCR. In fact, the primary target of diazepam is not a GPCR but are the ionotropic GABA_A_ receptors, which are known to influence the activity of chloride ion channel, hence inducing a tranquilizing effect^92,93^. Nevertheless, a recent study also suggested Benzodiazepine to interact with the Platelet-activating factor receptors^94^.

Given that these interactions were already listed in the GLASS database, at the best we can claim here is that our models can memorize correctly what they have encountered during the training process. Nevertheless, this gives confidence on the integrity of the data for entries listed in different databases. As expected, achieving high chemical structure fidelity using widely used file formats to store chemical structure information, such as the Simplified Molecular Input Line Entry Specification (SMILES) is not always possible^95^. Showing that the models could correctly annotate ligands retrieved from different databases is a hallmark for the data integrity in these databases (i.e. GLASS & Drug-Bank).

In subsequent studies, we are planning to build on the results obtained from this study and extend them by including other algorithms and representation methods. Additionally, we will focus on specific GPCR target classes and perform more rigorous QSAR analysis to understand the critical chemical motifs responsible for exerting the observed biological activities. Agonistic & partial agonistic, antagonistic and partial antagonistic activities will be also explored. These levels of detail are not very meaningful when working with a multi-class classification problem with many targets. Researchers are encouraged to use the prediction output from our models to discover additional potential repurposing opportunities, as we lack the required resources to pursue all of them.

### Study limitations

Despite the excellent performance and predictive power of our models, there are still limitations that need to be overcome in follow up studies by our group or other interested researchers. First, the quality of the GLASS dataset is not perfect and the data curation process in GLASS is partially automated, which can possibly lead to unexpected errors. Although GLASS may be the largest available repository for annotated GPCR ligands, other more meticulously curated databases can be exploited. Furthermore, due to the lack of sufficient training instances for many of the GPCR sub-types, we were urged to collapse many of these sub-types into a single GPCR parent class. Therefore, it may be important to follow up on predictions made by our models through more specialized models to decide the exact GPCR sub-type, provided that sufficient data are available to train these models. In an ideal scenario, several models can be developed, stacked in a hierarchical fashion until the final prediction (exact GPCR type/sub-type) can be identified.

Additionally, one may question our approach of using only the GLASS records for ligands showing the highest reported activities, and omitting other entries where the same ligand is active against other targets. From our perspective, this step was mandatory to reduce record’s ambiguity. The data curation protocol in GLASS is partially automated through text mining approaches^29^, and unlike other databases (e.g., Opentargets and CHEMBL), GLASS lacks confidence scores, which should help to select the best interaction records to be used in rigorous modeling. An example of studies that have followed the approach of selecting records with best confidence scores is the one conducted by Peon et al.^28^. Another example is the STITCH database, which considers only interactions with the strongest reported activity in building the dataset and in calculating final scores to reduce records’ ambiguities^96^. Therefore, we decided to focus only on ligand instances with the highest activities, which we believe should provide the most specific target predictions. In the Supplementary Information S5, we have reported a comparison between our approach (duplicate ligand records removed) and another approach where no duplicate ligands records were removed. The validation scores showed, without exception, that all models trained using the ECFP6 fingerprints or RDKit descriptors consistently performed better with our original approach (i.e. duplicate ligand records removed). More systematic analyses to tackle this limitation are to follow in future studies, such as performing phylogenetic analyses and possibly consensus interactions will be attempted.

Another problem is the dataset imbalance problem and it can be argued that such imbalance may reduce the accuracy of the developed models. Naturally, not all GPCRs are therapeutically important, and the amount of data available for a given GPCR is directly proportional to its therapeutic relevance. To overcome this limitation, we tried the following approach. First, we deliberately removed GPCR targets where the corresponding assay records are under-represented within GLASS (< 25 assay records, arbitrary cut-off). Second, we calculated performance measures that are well known by their robustness against the class imbalance problem, particularly the MCC scores.

Indeed, even with these limitations, we could achieve a performance that exceeds 90% on the test set. We preferred not to exploit methods that do under-sampling, over-sampling or those that can generate artificial data points. This was mainly because of two reasons. First, it was clear from the performance scores that the models performed very well. Second, it was not obvious how these methods, particularly data augmentation methods (e.g. SMOTE), can handle known QSAR issues, such as activity cliffs^97–99^. Data augmentation methods generally work by creating artificial observations (i.e. hypothetical molecules) through introducing a small level of noise to the adopted data representation techniques^100^. Although this may be understood in other fields of AI, such as computer vision where an image can be slightly perturbed (e.g. rotated, stretched, etc.) before the fitting process without a significant impact on the target class, in QSAR analysis, however, this behavior is not absolutely guaranteed.

Because of activity cliffs, even a small perturbation of a given chemical structure (e.g. indirectly through perturbing the molecular properties/descriptors space) can cause a huge difference in the biological activity. Sometimes tiny structural modifications can abolish or even reverse the biological activities of the small molecules against a given biological target^101,102^. Furthermore, to the best of our knowledge, the majority of studies that reported the use of modified sampling in QSAR analysis were for binary, not, multi-class classification problems^103,104^. Therefore, we are still hesitant to advocate the imperceptive applications of these techniques in the machine learning for QSAR or the drug discovery field in general, unless a full systematic analysis of its impact on the quality of the generated models is quantified. We are further enhancing the predictive power of the models and we may offer them as an online service for the research community. Currently, the models will be deposited on github together with a prediction python script and sample input and output files. The github repository (https://github.com/mmagithub/GPCR_LigandClassify) is available under the MIT license agreement for non-commercial purposes. Our lab is open for potential collaboration. Commercial users should contact us for further discussion.

Finally, we hope that the data and level of success presented in the current manuscript can inspire other researchers to tackle similar problems to quickly find and repurpose life-saving therapies for some pressing human threats. For many of these threats, the typical drug discovery life cycle is worthless. An example of these threats includes the recent pandemic outbreak of the deadly Coronavirus disease (COVID-19).

## Supplementary Information

Supplementary Information 1.

Supplementary Information 2.

Supplementary Information 3.

Supplementary Information 4.

Supplementary Information 5.

Supplementary Information 6.

## Data Availability

The models, a running script and sample inputs & output files are available through github free of charges for non-commercial users, check: https://github.com/mmagithub/GPCR_LigandClassify
