# Supplementary figures and images for: GPCR_LigandClassify.py; a rigorous machine learning classifier for GPCR targeting compounds
Source: Sci Rep. 2021 May 4;11:9510. doi: 10.1038/s41598-021-88939-5 (PMC8097070; doi:10.1038/s41598-021-88939-5)

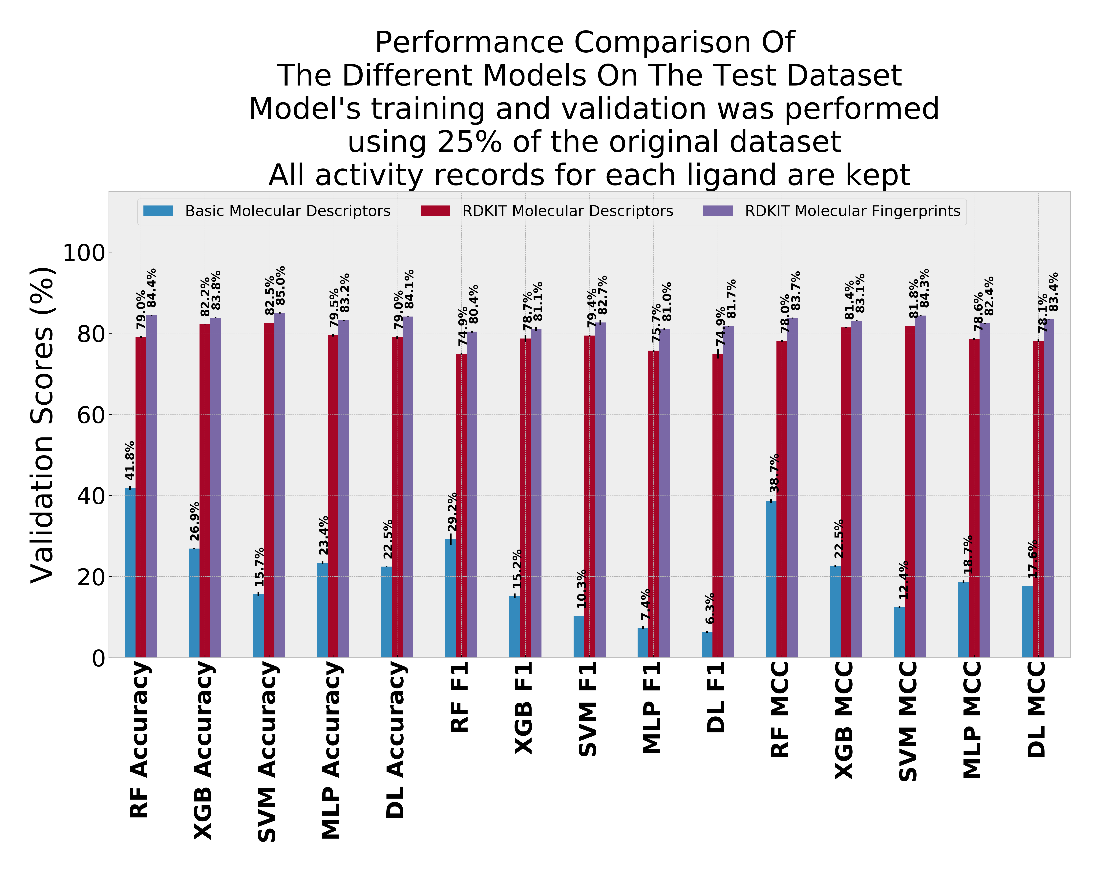


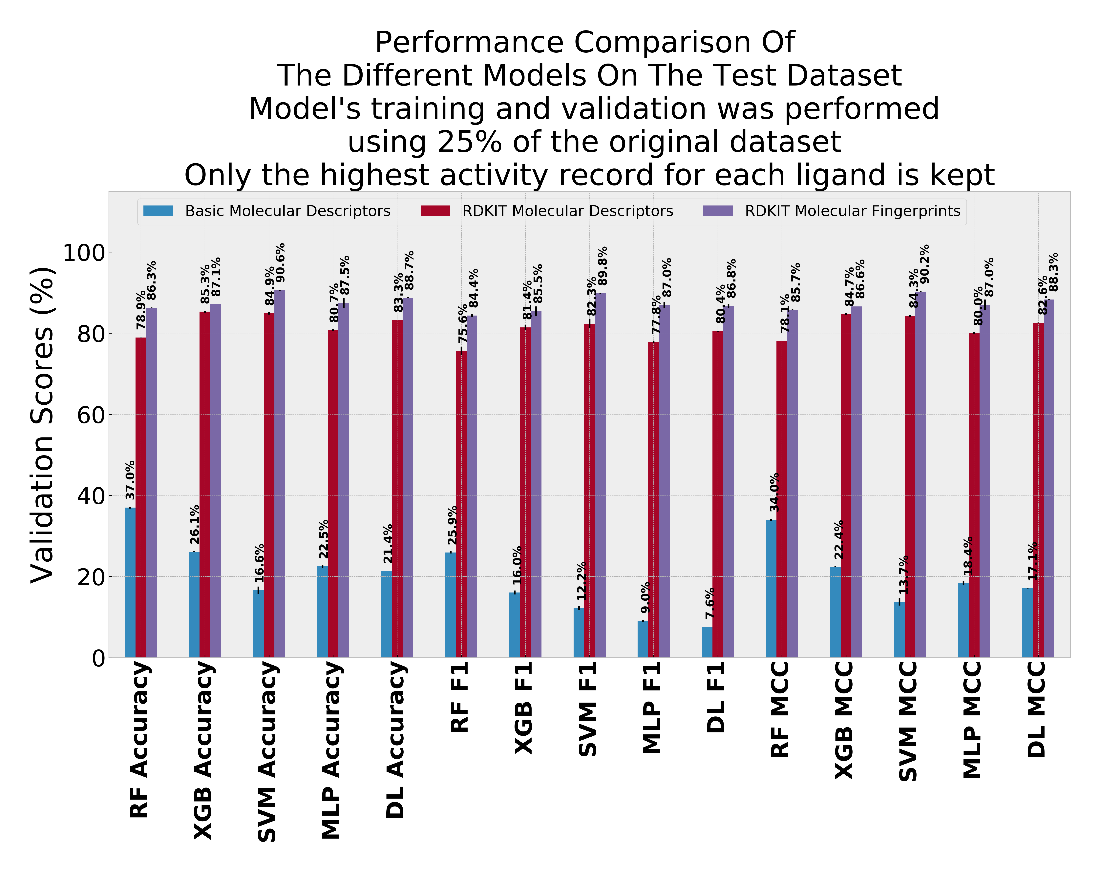

Supplement: Supplementary file 5 — Supplementary Information 5. [file 41598_2021_88939_MOESM5_ESM.docx]
