# Supplementary material for: GPCR_LigandClassify.py; a rigorous machine learning classifier for GPCR targeting compounds
Source: Sci Rep. 2021 May 4;11:9510. doi: 10.1038/s41598-021-88939-5 (PMC8097070; doi:10.1038/s41598-021-88939-5)
Supplement: Supplementary file 6 — Supplementary Information 6. [file 41598_2021_88939_MOESM6_ESM.docx]

***Random Forest Classifier (RF)***

In general, RF algorithm is one of the best performing ML algorithms for tabular data ^1^. In its simplest definition, the RF algorithm is a classification (or regression) technique based on an ensemble of decision trees. Using bootstrapped samples of the training data and though a random selection of input features (descriptors, fingerprints, etc.…), each decision tree recursively split the data to form a homogenous subset of subclasses such that maximum information (low entropy) is gained at each tree split ^2^. As discussed in Polishchuk et. al. ^3^, in QSAR modeling, RF excels in three aspects; i) resistance to overfitting (to some extent though), ii) minimal requirement of hyperparameters tuning & iii) accounting for diversity eg., compounds with different mechanisms of action can be studied within the same dataset. The Random forest algorithm has been proven successful in several QSAR analyses for drug/drug-like molecules ^2^. For example, in a comparative study by Russo et al., which aimed at discovering the best performing prediction algorithm for estrogen receptor binding, RF was ranked as the top-performing algorithm, beating deep learning ^4^. For more details about the RF algorithm, readers are encouraged to consult the excellent resources cited within this section.

***Extreme Gradient Boosting Classifier (XGBoost)***

On the contrary to the RF algorithm, which trains the ensemble of decision trees in a “bagging” or a parallel manner, other decision tree ensemble methods train the decision trees in a sequential, additive strategy or “boosting”. In boosting, the newly added learner (a decision tree) attempts to minimize the error generated from the previous learners.

Presumably, the most popular “boosting” algorithm is the Extreme Gradient Boosting (XGBoost) algorithm ^5-7^. XGBoost exploit several regularization tricks to overcome “over-fitting” which is a common problem with other decision tree ensemble algorithms, including Random Forests. The XGBoost algorithm has gained tremendous popularity in QSAR modeling research given its training speed and robustness ^5-9^ in recent years. With respect to speed, recent benchmarks for XGBoost against other resource-intensive ML models showed that XGBoost, with less computational resources, offers a similar (sometimes better) overall performance relative to deep learning or RF. XGBoost can consume less than third of the time required by deep learning or RF, even on a single CPU ^8^.

***Support Vector Machines (SVMs)***

Although ensemble decision-trees based algorithms, including RF & XGBoost, have greatly replaced the applications of support vector machines in many applications, in QSAR modeling, SVM is still one of the best performing and widely used ML algorithms ^10^. In SVM, a kernel function tries to find the maximum separation hyper-plane between different classes in the high-dimensional space of input features. SVM has been very popular in QSAR modeling for studying drug-induced toxicity ^11^, drug binding ^12^, virtual screening ^13^ as well as in the prediction of physicochemical properties of drug-like organic molecules ^14^. SVM is particularly powerful in high-dimensional space problems. A distinct advantage of SVM is its generalization performance that makes SVM more resistant to overfitting compared to other ML algorithms ^10^.

***Artificial Neural Network (ANN): Multi-Layer Perceptron (MLP) & Deep Neural Network (DNN)***

Recently, the application of artificial neural networks (ANNs) in QSAR modeling has pushed the field to an entirely new level ^15^. Given their capacity to handle the extreme level of noise that usually accompanies experimental biochemical and biological measurements, it is not surprising that ANNs have had a great surge in QSAR studies in the last few years. With enough training samples and the careful selection of the model’s architecture and hyper-parameters, the predictive power of ANNs models can surpass even the most sophisticated ML algorithms in the majority of the cases. Taken together, ANNs based modeling is an ideal choice for the field of QSAR analysis of drug/drug-like molecules.

In the field of QSAR/QSPR modeling, the simplest form of a supervised ANN function is to map a set of input features (molecular descriptors or fingerprints) to an output, say a biological activity, through optimizing a set of weights assigned to each input feature ^16,17^. Non-linearity is introduced by special mathematical functions (activation functions). The prediction error is minimized through an iterative process called back-propagation. Each ANN mapping function is called a node and is arranged in layers of multiple nodes (or a perceptron), and is connected to nodes in other layers in a manner that is architecture-dependent. Technically speaking, any feed-forward ANN architecture, the proto-type of ANN architecture, should consist of at least two layers, an input layer and an output layer of ANN nodes with any number of hidden layers exist between these two layers ^18^. Depending on the number of layers, the feed-forward architecture can be either shallow (no hidden layers) learning or deep (one or more hidden layers) learning. Given its exceptional predictive power, deep learning has been extensively used in QSAR modeling, for virtual compound screening ^19^, multi-target drug discovery ^20^, toxicity modeling ^21^ and more.

In the current study, we used two different implementations of ANNs, the Multi-Layer Perceptron Classifier (MLP-Classifier) as implemented in Scikit-Learn and the Deep Learning Classifier (DNN-Classifier) as implemented in Tensor-flow. We did not use any hidden layers for the MLP-Classifier (i.e. a shallow learning model was generated), whereas a single hidden layer was used for the DNNClassifier in Tensor-flow (i.e. a deep learning model was generated).^15^

**References**

1. Breiman, L. Random forests. *Machine learning* **45**, 5-32 (2001).

2. Svetnik, V.*, et al.* Random forest: a classification and regression tool for compound classification and QSAR modeling. *J Chem Inf Comput Sci* **43**, 1947-1958 (2003).

3. Polishchuk, P.G.*, et al.* Application of random forest approach to QSAR prediction of aquatic toxicity. *J Chem Inf Model* **49**, 2481-2488 (2009).

4. Russo, D.P., Zorn, K.M., Clark, A.M., Zhu, H. & Ekins, S. Comparing Multiple Machine Learning Algorithms and Metrics for Estrogen Receptor Binding Prediction. *Mol Pharm* **15**, 4361-4370 (2018).

5. Chen, T. & Guestrin, C. Xgboost: A scalable tree boosting system. in *Proceedings of the 22nd acm sigkdd international conference on knowledge discovery and data mining* 785-794 (ACM, 2016).

6. Chen, T. & Guestrin, C. XGBoost: reliable large-scale tree boosting system. arXiv. *2016a. ISSN*, 0146-4833 (2016).

7. Babajide Mustapha, I. & Saeed, F. Bioactive Molecule Prediction Using Extreme Gradient Boosting. *Molecules* **21**(2016).

8. Sheridan, R.P., Wang, W.M., Liaw, A., Ma, J. & Gifford, E.M. Extreme Gradient Boosting as a Method for Quantitative Structure-Activity Relationships. *J Chem Inf Model* **56**, 2353-2360 (2016).

9. Wacker, S. & Noskov, S.Y. Performance of Machine Learning Algorithms for Qualitative and Quantitative Prediction Drug Blockade of hERG1 channel. *Comput Toxicol* **6**, 55-63 (2018).

10. Mei, H., Zhou, Y., Liang, G. & Li, Z. Support vector machine applied in QSAR modelling. *Chinese Science Bulletin* **50**, 2291-2296 (2005).

11. Tharwat, A., Moemen, Y.S. & Hassanien, A.E. Classification of toxicity effects of biotransformed hepatic drugs using whale optimized support vector machines. *J Biomed Inform* **68**, 132-149 (2017).

12. Li, H.*, et al.* Prediction of estrogen receptor agonists and characterization of associated molecular descriptors by statistical learning methods. *J Mol Graph Model* **25**, 313-323 (2006).

13. Bushdid, C., de March, C.A., Fiorucci, S., Matsunami, H. & Golebiowski, J. Agonists of G-Protein-Coupled Odorant Receptors Are Predicted from Chemical Features. *J Phys Chem Lett* **9**, 2235-2240 (2018).

14. Harding, A.P., Wedge, D.C. & Popelier, P.L. pK(a) prediction from "Quantum Chemical Topology" descriptors. *J Chem Inf Model* **49**, 1914-1924 (2009).

15. Baskin, II, Palyulin, V.A. & Zefirov, N.S. Neural networks in building QSAR models. *Methods Mol Biol* **458**, 137-158 (2008).

16. Ghasemi, F., Mehridehnavi, A., Perez-Garrido, A. & Perez-Sanchez, H. Neural network and deep-learning algorithms used in QSAR studies: merits and drawbacks. *Drug Discov Today* **23**, 1784-1790 (2018).

17. Gonzalez-Arjona, D., Lopez-Perez, G. & Gustavo Gonzalez, A. Non-linear QSAR modeling by using multilayer perceptron feedforward neural networks trained by back-propagation. *Talanta* **56**, 79-90 (2002).

18. Svozil, D., Kvasnicka, V. & Pospichal, J. Introduction to multi-layer feed-forward neural networks. *Chemometrics and intelligent laboratory systems* **39**, 43-62 (1997).

19. Myint, K.-Z., Wang, L., Tong, Q. & Xie, X.-Q. Molecular fingerprint-based artificial neural networks QSAR for ligand biological activity predictions. *Molecular pharmaceutics* **9**, 2912-2923 (2012).

20. Ajmani, S. & N Viswanadhan, V. A neural network-based QSAR approach for exploration of diverse multi-tyrosine kinase inhibitors and its comparison with a fragment-based approach. *Current computer-aided drug design* **9**, 482-490 (2013).

21. Fjodorova, N., Vračko, M., Jezierska, A. & Novič, M. Counter propagation artificial neural network categorical models for prediction of carcinogenicity for non-congeneric chemicals. *SAR and QSAR in Environmental Research* **21**, 57-75 (2010).
